# Unlocking the potential of informal healthcare providers in tuberculosis care: insights from India

**DOI:** 10.1136/bmjgh-2024-015212

**Published:** 2024-02-26

**Authors:** Poshan Thapa, Padmanesan Narasimhan, Kristen Beek, John J Hall, Rohan Jayasuriya, Partha Sarathi Mukherjee, Surbhi Sheokand, Petra Heitkamp, Prachi Shukla, Joel Shyam Klinton, Vijayshree Yellappa, Nitin Mudgal, Madhukar Pai

**Affiliations:** 1 School of Population and Global Health, McGill University, Montreal, Quebec, Canada; 2 TB-PPM Learning Network, McGill International TB Centre, McGill University, Montreal, Quebec, Canada; 3 School of Population Health, University of New South Wales, Sydney, New South Wales, Australia; 4 Liver Foundation, Kolkata, West Bengal, India; 5 Research Institute of the McGill University Health Centre, Montreal, Quebec, Canada; 6 World Health Partners, New Delhi, Delhi, India

**Keywords:** Global Health, Health systems, Public Health, India, Tuberculosis

In 2022, tuberculosis (TB) remained a major global health concern, second only to COVID-19 in mortality from a single infectious agent. Over 10 million people contract TB annually, with two-thirds of cases from eight high-burden countries. India alone accounted for 27% of the global burden, totalling an estimated 2.8 million cases.^1^ Notably, approximately 18% of these people were considered ‘missing’, either undiagnosed or not reported, because they were likely managed by the private sector, which serves the healthcare needs of about half of the patients with TB in the country. The private health sector in India, which delivers approximately 87% (in some regions, particularly if underserved) of initial primary care, is diverse and largely unregulated, extending from small clinics to multispecialty hospitals and ranging from informal providers to highly qualified specialists.^2^ This poses significant challenges, as patients seeking care from this sector often experience delayed TB diagnoses and inappropriate treatments.^3^ Therefore, to enhance TB care access and quality, it is essential to involve all healthcare providers in the private sector, both formal and informal, within the framework of the Public-Private Mix, as recommended by India’s National Strategic Plan (NSP) for TB elimination (2017–2025).

A significant yet underutilised group within the private sector are informal healthcare providers (IPs), who, despite the NSP’s inclusive approach, remain largely underprioritised in the National TB Elimination Programme (NTEP) of India. These IPs, often known as rural medical practitioners or village doctors, typically operate outside the formal health system and lack accredited qualifications, often dispensing allopathic treatments such as antibiotics and injections without formal training.^4 5^ As in India, IPs are prevalent in many other low-income and middle-income countries (for instance, 65% of primary care in Bangladesh and 77% in Uganda is provided by IPs), who often serve as the first point of healthcare contact in communities.^4 6 7^ Community health workers do not fall under the category of IPs, as they are typically trained and integrated into the formal health system or non-governmental organisations. Also, IPs are distinct from alternative providers such as the AYUSH (Ayurveda, Yoga and Naturopathy, Unani, Siddha and Homeopathy) system in India, who are usually trained in accredited institutions and are part of a formally recognised system of medicine.

In this editorial, we explore the critical role and importance of IPs in TB care. We seek to offer a nuanced understanding of the roles IPs can assume and suggest strategies for their effective integration into TB care. By focusing on these providers, we aim to shed light on an overlooked aspect of India’s effort to combat TB. While our editorial is focused on India, the insights and approaches we present are applicable to other countries with large and diverse private health sectors and a high TB burden, such as Pakistan, Bangladesh, Indonesia, Nigeria, the Philippines and the Democratic Republic of the Congo.

## Significance of IPs in TB care

The importance of IPs in TB care is rooted in their widespread presence and strong acceptance within communities, where they frequently serve as the initial point of contact for patients seeking healthcare.^4^ The prevalence of IPs in India’s health system landscape is evident from the WHO’s India Health Workforce Report.^8^ This report indicates a significant portion of those identified as allopathic doctors lack formal medical training: 31.4% have only secondary school education, and an even more notable, 57.3% do not possess medical qualifications. These figures align with findings from a National Sample Survey-based study by Rao *et al*, which found that 42.3% of doctors (identified locally) across various regions in India did not meet the recommended qualifications.^9^ Similar trends are also evident in other regional surveys conducted throughout India.^10–12^

In rural and underserved areas, the significance of IPs in primary healthcare is particularly pronounced, as captured in our qualitative research.^13^ These regions, often facing a higher TB burden, rely heavily on IPs for primary care services. Individuals with TB symptoms, such as cough, often seek initial care from community-based providers such as IPs and pharmacists. This trend is notable, as IPs have been reported to be the first or preferred healthcare option for a substantial percentage of patients with TB as documented in health-seeking behaviour studies conducted in different regions of India.^14 15^ Such early interaction of people seeking care with IPs is important, as it provides an invaluable opportunity for the early detection and referral of people with TB in these high-need areas, where IPs serve as the cornerstone of healthcare.

## A closer look at IPs’ TB care knowledge and practices

An analysis of the existing evidence reveals a disconnect between practice and policy concerning IPs in TB care. Despite the lack of clear policy guidance on the role and engagement of IPs in NTEP, studies indicate that they are actively involved in providing TB care at the primary level. For instance, a study from rural Haryana found that 54% of IPs were consulted by 2–5 patients with TB monthly.^16^ Another study revealed that some IPs retained and treated patients for 3–4 months with TB drugs before making an appropriate referral.^15^ Our team’s survey of 203 IPs in West Bengal further highlighted this reality.^17^ It showed that IPs saw, on average, five patients with TB symptoms every 6 months, with two of them typically confirmed to have TB. This survey also exposed certain concerning practices among IPs, such as delayed referrals (only 34% referred during the first visit) and overuse of antibiotics (as high as 69% during the first visit), contributing to the diagnostic delays we observed among patients with TB in our separate study.^18^

Moreover, our research in India, employing methods like standardised patients, has identified variable and often suboptimal quality of TB care among private providers, including IPs.^19^ In a separate study by our team involving 331 IPs, we found low competence in history-taking, indicating their limited ability to effectively screen presumptive TB cases.^20^ This finding is particularly significant given IPs’ role as primary care providers in communities. Collectively, these findings stress the urgent need for targeted interventions aiming to enhance the TB care knowledge and practices of IPs and align them with India’s national TB care standards.

## Potential of improved outcomes by engaging IPs in TB care

Importantly, the accumulating body of evidence demonstrates the untapped potential of IPs to significantly improve TB care outcomes. Studies conducted in diverse settings, including Bangladesh, Malawi and India, have reported improved TB testing, case notification and treatment outcomes when IPs are engaged and supported.^21–24^ These consistent findings affirm the positive impact that IPs can have on TB care. Complementing these results, our team’s scoping review has documented the positive impacts of involving IPs across multiple domains of TB care (from prevention and detection to treatment).^25^ Additionally, the value of training IPs has been highlighted by several studies, including a randomised controlled trial in India, which reported a 14.2% improvement in the correct management of various conditions by IPs.^20 24 26^ Given this evidence, it is clear that prioritising and integrating IPs in TB care programmes is not just beneficial but necessary for enhancing care outcomes.

## Recommendations for advancing the role of IPs in TB care

**Figure 1:**
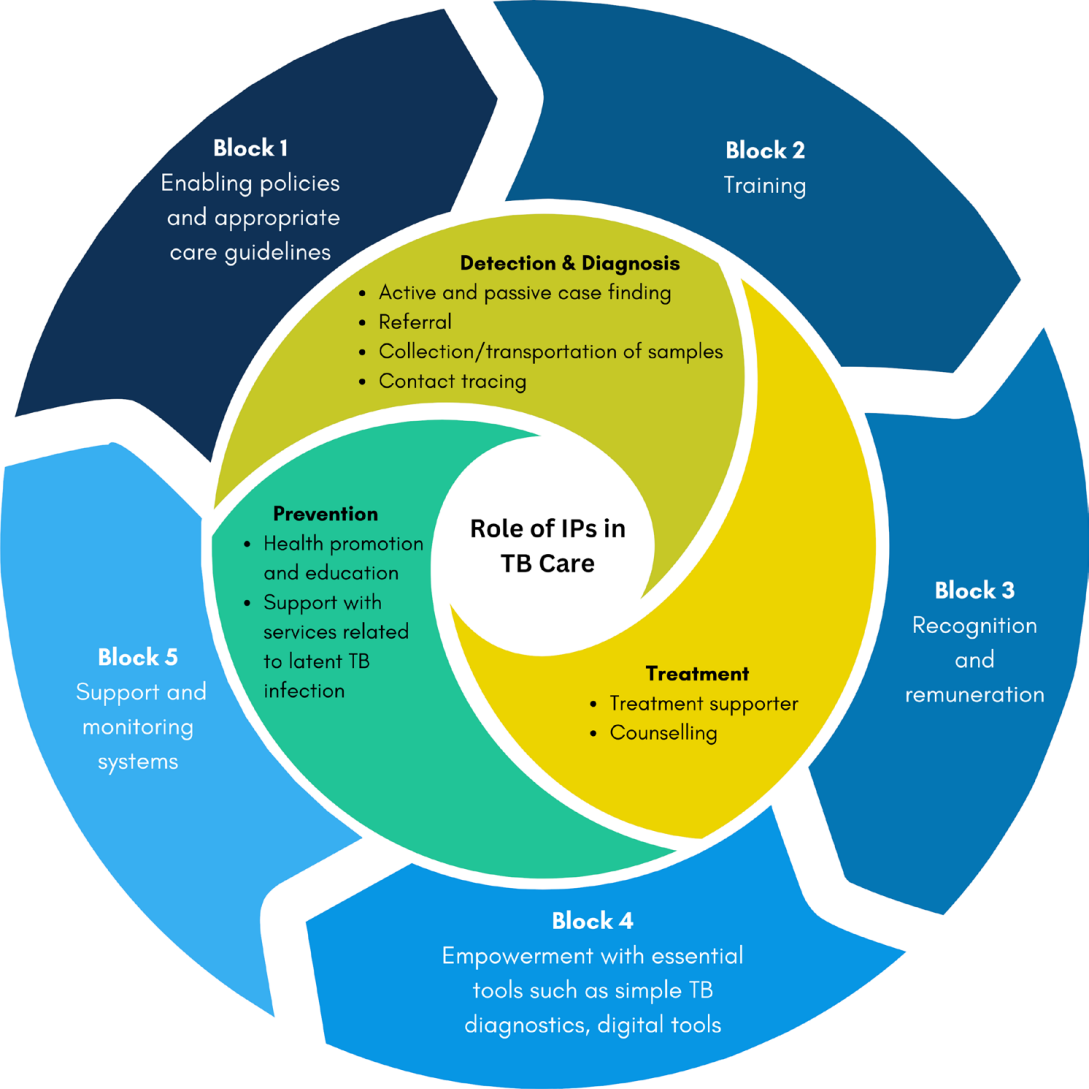
Roles and foundational building blocks for IPs in TB care. IPs, informal healthcare providers; TB, tuberculosis.

In figure 1, we illustrate the diverse roles that IPs can assume in TB care, as well as the essential elements needed for their engagement, depicted as foundational building blocks.

The roles of IPs in TB care, as illustrated in figure 1, are synthesised from findings derived from our comprehensive scoping review and quantitative and qualitative studies.^13 17 25 27^ According to the International Standards for TB care,^28^ these roles can be undertaken by non-medical professionals, given appropriate training, a stance supported by existing literature that associates IP engagement with improved care outcomes.

To optimise IP engagement in these critical TB care functions, it is essential to consider five prerequisite foundational building blocks.

### Block 1: enabling policies and appropriate care guidelines

Clear policies are needed to guide IP engagement within National TB Programmes (NTPs), providing clarity on roles and expectations and facilitating the development of national guidelines, such as protocols for screening and referral of presumptive TB cases by IPs. Moreover, to ensure the responsible management of TB care at the community level, policies must be clearly outlined to restrict IPs from prescribing anti-TB medications. Furthermore, educating IPs about the WHO’s AWaRe (Access, Watch and Reserve) framework is imperative to tackle the misuse of critical antibiotics.^29^

### Block 2: training

Structured training programmes are essential to improve IPs’ TB care knowledge and align their practices with national guidelines. The benefits of training IPs are well documented.^26^

### Block 3: recognition and remuneration

Recognising IPs’ contributions to health systems is vital, as seen, for example, in the initiatives by the state government of West Bengal in India. This step has led to IPs’ expanded engagement in formal programmes, including those addressing the COVID-19 pandemic.^13^ IPs should also be provided financial incentives for their TB care work, integrated into the existing government incentive system within NTEP.

### Block 4: empowerment of IPs with essential tools such as simple TB diagnostics and digital tools

Empowering IPs with digital tools, like screening applications, and point-of-care diagnostic tools, such as tongue swab-based TB tests and sputum sample collection kits, can unlock significant potential for early case detection and referral.^30^ This area holds promise for future implementation research.

### Block 5: support and monitoring systems

Support and monitoring systems, akin to those for the broader private sector, can be implemented for IPs through the development of tools, monitoring mechanisms and capacity-building interventions, as discussed in our research involving IPs and formal practitioners in the NTEP.^13^

A system-level approach is required to recognise the critical role of IPs, a principle that extends beyond TB care. Their inclusion in TB care can serve as a model for broader healthcare system engagement. IPs are a valuable resource to address health workforce shortages, and their inclusion in NTEP exemplifies the concept of task-shifting, where TB services are delivered by preferred community providers, ensuring timely referrals and adherence to national standards. Integrating IPs into the healthcare framework contributes to TB care enhancement and is a step towards improving equitable healthcare access, especially in underserved areas.

In summary, we have highlighted the significant role of IPs in TB care, emphasising their potential to improve TB care outcomes. Supported by a growing body of evidence, we argued for the need for clear policies, training, recognition and incentives, including diagnostic and digital tools, to harness the full potential of IPs in TB care. We advocated for their inclusion as essential providers of TB care, setting an example for broader engagement in health systems. This approach is particularly beneficial in underserved and impoverished regions, extending beyond India to other countries facing high TB burdens and characterised by large, unregulated private health sectors.

## Data Availability

All data relevant to the study are included in the article.
